# Professional wellbeing and turnover intention among child therapists: a comparison between therapists trained and untrained in Trauma-Focused Cognitive Behavioral Therapy

**DOI:** 10.1186/s12913-022-08670-3

**Published:** 2022-11-08

**Authors:** Samira Aminihajibashi, Ane-Marthe Solheim Skar, Tine K. Jensen

**Affiliations:** 1 Norwegian Centre for Violence and Traumatic Stress Studies, Hausmanns Gate 3, NO-0186 Oslo, Norway; 2 Department of Psychology, University of Oslo, Oslo, Norway

**Keywords:** Burnout, Secondary traumatic stress, Compassion satisfaction, Professional quality of life, Occupational health

## Abstract

**Background:**

Poor professional wellbeing and job turnover is challenging for child mental health clinics and despite an increasing interest in implementing evidence-based practices (EBPs) in mental health services, little is known about if and how using EBPs may influence therapists’ professional wellbeing and turnover intention. To investigate this, we compare the average level of compassion satisfaction, burnout, secondary traumatic stress, and turnover intention between therapists trained in an EBP (Trauma-Focused Cognitive Behavioral Therapy – TF-CBT) and untrained therapists. We also explore the prevalence of and the associations between these personal and organizational outcomes.

**Method:**

In this cross-sectional study, the data is collected from a national sample of 373 therapists 5 years after an implementation program began (i.e., in the sustainment phase). The variables were measured by the Professional Quality of Life and the Turnover Intention Scales. The Evidence-Based Practice Attitude Scale was also used to measure therapists’ attitudes toward EBPs.

**Results:**

Over 70% of the respondents reported medium to high levels of burnout, secondary traumatic stress symptoms, and compassion satisfaction, whereas one-third of the respondents reported a high level of intention to leave their job in the current or near future. Higher ratings on burnout and secondary traumatic stress were significantly associated with lower compassion satisfaction and higher turnover intention. Finally, we found significantly lower degree of burnout and turnover intention along with higher compassion satisfaction among TF-CBT therapists (*n* = 96), compared to other therapists who were not trained in TF-CBT (*n* = 231). These differences could not be explained by between-group differences in age, job tenure, educational background, or therapists’ attitudes towards EBPs. However, mean differences in ratings on secondary traumatic stress symptoms were not statistically significant.

**Conclusion:**

Although the prevalence findings are in general alarming, the present study provides the first empirical evidence for a potential positive effect of being trained in TF-CBT on therapists’ wellbeing and turnover intention. We discuss these findings in the light of self-efficacy theory and the job demands-resources model.

## Background

Treating traumatized youth can be both challenging and rewarding. Compassion satisfaction (i.e., the perceived pleasure experienced from helping others, receiving collegiate support, and doing a good job) and compassion fatigue (i.e., the reduced ability to empathize) refer, respectively, to the positive and negative outcomes of therapy work. Compassion fatigue can consist of symptoms of burnout (i.e., feelings of hopelessness, anger, frustration, depression, and reduced perceived self-efficiency), and secondary traumatic stress (i.e., symptoms similar to post-traumatic stress disorder (PTSD) such as re-experiencing other people’s detailed description of traumatic events, having invasive thoughts, or being hypervigilant) [1]. Perceived levels of compassion satisfaction, burnout, and secondary traumatic stress can be considered to be subjective measures of professional quality of life [1], and workplace wellbeing [2–4].

Poor workplace wellbeing can negatively affect therapists’ physical health and their personal and professional relationships [5–7], and lead to an increase in turnover intention, i.e., a deliberate willfulness to leave the current organization, which is a reliable determinant of actual turnover behavior [8–10]. High staff turnover is an ongoing concern in mental health services because recruitment and training of new staff are costly and because it interferes with the care quality and the sustainability of implementation programs, especially in the sustainment phase, i.e., approximately 5 years after initiating the implementation [11–14]. However, the scope of compassion satisfaction and turnover *intention* among child therapists is understudied. Also, despite an increasing interest in implementing evidence-based practices (EBPs) in Child and Adolescent Mental Health Services (CAMHS) [15, 16], studies on the impact of using EBPs on workforce wellbeing and turnover intention are limited and the findings are contradictory.

In this cross-sectional study, we aim to fill these gaps and expand the literature. We first explore the prevalence of and the associations between compassion satisfaction, burnout, secondary traumatic stress, and turnover intention in a national sample of child therapists. Second, we examine whether therapists trained in TF-CBT report lower ratings of burnout, secondary traumatic stress, and turnover intention along with higher compassion satisfaction in the sustainment phase of an implementation program compared to a comparable group of therapists who are not trained in TF-CBT (comparison group). TF-CBT is the treatment of choice for treating trauma in children and is being widely implemented [17]. For this reason it is of particular interest to study possible effects of being trained in this program.

### Prevalence of wellbeing measures and turnover intention among child therapists

Research findings have frequently revealed a medium to high level of secondary traumatic stress and burnout among mental health providers [18, 19] including child therapists [20]. Working with traumatized children can be particularly emotionally challenging and increase the risk of burnout [21] and compassion fatigue [22, 23]. A recent study revealed for instance that 75% of psychiatrists working in Irish CAMHS experienced moderate to high levels of burnout, and 70% expressed a wished to change their job [24]. To the best of our knowledge, this is the only study that has explored the turnover *intention* rate among therapists working in CAMHS. Knowledge on the prevalence and predictors of child therapists’ turnover *intention* may prevent staff turnover if adequate measurements are taken.

On the other hand, some studies have found that the majority of mental health professionals also report a medium to a high level of compassion satisfaction [18, 25, 26]. In other words, although measures of burnout and secondary traumatic stress usually associate positively with each other, and negatively with compassion satisfaction [18, 27, 28], these measures of positive and negative wellbeing are independent concepts and one can experience a high level of both [29]. To the best of our knowledge, the scope of compassion satisfaction and its association with turnover *intention* among child therapists has not been studied, although some studies have found that a high level of *job* satisfaction is associated with reduced turnover intention among therapists working with traumatized adults [30, 31]. Since compassion satisfaction may act as a protective factor against both burnout and secondary traumatic stress [32, 33], knowledge on the prevalence and predictors of compassion satisfaction can improve both therapists’ professional wellbeing and the provided care quality.

### Effect of using EBPs on therapists’ wellbeing measures and turnover intention

Findings from previous studies suggest that some factors like being older, having longer work experiences [18, 25, 34], and using EBPs [9, 25, 35] can be shared predictors of both compassion fatigue and compassion satisfaction, as well as staff turnover. A recent review article revealed that the association between negative outcomes of working with trauma can be influenced by several factors including using EBPs [36, 37].

Since implementing EBPs requires additional time-demanding activities for training, supervision, and fidelity monitoring, using EBPs can pose some challenges and increase the level of stress, emotional exhaustion, and turnover rates in therapists [38, 39]. However, other studies have revealed that using EBPs can be positively associated with compassion satisfaction, and negatively with burnout and staff turnover in child welfare [9, 35] and adult mental health services [40, 41].

Within the field of trauma therapy, we found only two studies, both from the United States that investigated the effect of using EBPs on the wellbeing of therapists. Kim and colleagues [11] studied predictors of burnout among therapists working in community children’s mental health agencies in which six EBPs were implemented. They found that while therapists’ theoretical orientation and the amount of time that was allocated to the EBP implementation activities were not associated with burnout, the caseload, and the number of delivered EBPs predicted the risk of burnout. In contrast, Craig and Sprang [25], found that a higher extent of applying EBPs was associated with reduced risk of burnout and compassion fatigue, along with higher compassion satisfaction among trauma therapists who mainly worked with traumatized adults (80%).

Such discrepancies in findings can be related to the characteristics of both therapists and EBPs [42–44]. Some research findings indicate that using EBPs that consist of prescribed sessions and mandatory follow-up consultation, as well as being positive and open toward EBPs are associated with reduced emotional exhaustion and turnover among therapists working in children’s mental health services [11, 45].

### Self-efficacy theory and the job demands-resources model

Positive outcomes of utilizing EBPs can be understood in light of the self-efficacy theory and the job demands-resources model. Based on self-efficacy theory [46, 47], people´s beliefs in their ability to achieve desired outcomes through learning and executing required actions have a determining influence on their motivation, choices, behaviors, and, consequently, their personal and professional wellbeing. Since training and specialization in EBPs can enhance therapists´ skills and perceived self-efficacy in achieving their professional goals, one can expect that utilizing EBPs will have positive effects on therapists’ wellbeing and retention [11, 43].

Similarly, based on the job demands-resources (JD-R) model [48]), negative and positive professional outcomes depend on the balance between job demands (i.e., any demanding characteristics of a job that can cause strain) and job resources (i.e., any aspect of a job that is motivating and beneficial to reduce job demands and/or to achieve work goals and personal growth). This model suggests that job resources like access to work-related training can either buffer the negative effects of job demands on job strain [33], or mediate the relationship between job resources, on the one hand, and emotional exhaustion, work engagement, and turnover intention, on the other hand [48, 49]. Therefore, one can expect that training in EBPs will have positive effects on therapists’ wellbeing and retention intention.

### Present study

To the best of our knowledge, the impact of being trained in TF-CBT on clinicians’ professional quality of life and turnover intention is unknown. TF-CBT is the most well-researched and recommended EBP for treating child PTSD [17, 50, 51]. While the majority of available EBPs for childhood psychiatric disorders are rarely found in public mental health settings, TF-CBT has been widely disseminated and proven effective both in western and low- and middle-income countries [42, 52, 53]. In a recent study, Becker-Haimes and colleagues [54] found that compared to other treatment models, training in TF-CBT was the strongest predictor of higher self-efficacy ratings. When implemented successfully, in addition to increasing therapy skills and sense of self-efficacy, training in TF-CBT can also provide other job resources like more job clarity through predefined and structured treatment sessions along with necessary boundaries, proper administrative support, and social support from devoted teams and supervisors that give constructive feedback [38, 43]. Considering these characteristics and research findings, and based on the self-efficacy theory, the job demands-resources model, and the abovementioned, one can expect that training in TF-CBT will have a positive effect on both the professional quality of life and turnover intention among therapists. All in all, based on the abovementioned literature, we hypothesized that:

- *Hypothesis 1.* Most therapists will report medium to high levels of both compassion fatigue (i.e., burnout and secondary traumatic stress) and compassion satisfaction.
- *Hypothesis 2.* Burnout, secondary traumatic stress, and turnover intention will be related positively to each other and negatively to compassion satisfaction, age, and job tenure.
- *Hypothesis 3.* On average, TF-CBT trained therapists will report lower ratings of burnout, secondary traumatic stress, and turnover intention along with higher compassion satisfaction than TF-CBT untrained therapists, even after controlling for the effect of therapists’ attitudes toward EBPs.

## Method

### Participants and procedure

All clinicians (*N* = 1241) working at the time of data collection (December 2017) in one of 84 Norwegian CAMHS were invited to participate through an online questionnaire. Two reminders were sent for no-responders. In Norway, CAMHS are the main specialist mental health services that provide free treatment for children and adolescents with relatively serious levels of mental health problems. Children and adolescents are required to have a diagnose or at least sub-threshold symptoms to receive treatment and the majority report exposures to potentially traumatizing events [55]. Around half of CAMHS participated in an implementation program, which involved training of all therapists in trauma and PTSD screening and training of a sub-sample of therapists in TF-CBT [56]. These CAMHS are hereafter referred to as TF-CBT CAMHS (*N* = 44). A total of 373 therapists (females = 310) responded to at least one questionnaire, indicating a response rate of 30%. A group of 256 therapists (females = 213, TF-CBT therapists = 92) responded to all questionnaires. A priori statistical power analysis was run using G*Power tool [57]. Results revealed that when power is set to 0.80, a minimum sample size of 64 is required per group (*N* = 128) in an independent samples t-tests to achieve a medium effect size, d = 0.5 [58], with an alpha level of 0.05, two-tailed. Thus, our sample is well in excess of the needed statistical power. Table 1 presents the demographics of the whole sample. Participants had different educational background, but almost half (*n* = 169) were psychologists. Females were in the majority (83%), and the median age was between 40 to 49 years (*n* = 127), but there was a broad age span from 20 to more than 60 years. The median job tenure was from 2 to 5 years, and around 50% (*n* = 185) have worked in the current CAMHS over 6 years. The majority (78%) of participants were from TF-CBT CAMHS whereas 32% of the therapists (*n* = 94) in these CAMHS were TF-CBT practitioners. All participants signed a digital consent form. The study received ethical approval from the Norwegian Regional Committee for Medical and Health Research Ethics. All methods were performed in accordance with the relevant guidelines and regulations.

**Table 1:** Sample Demographics (*N* = 373)

**Characteristics**	**Categories**	**Frequency (%)**
**Gender**	Females	310 (83.1%)
	Males	63 (16.9%)
**Age**	20—29	32 (8.6%)
	30—39	91 (24.4%)
	40—49	127 (34%)
	50—59	84 (22.5%)
	At least 60	39 (10.5%)
**Professional** **Background**	Psychologists	70 (18.8%)
	Psychology specialists	99 (26.5%)
	Medical doctors	18 (4.8%)
	Medical specialists	9 (2.4%)
	Psychiatrists	18 (4.8%)
	Child welfare officers	59 (15.8%)
	Social worker	45 (12.1%)
	Family therapist	18 (4.8%)
	Nurse	16 (4.3%)
	Social educator	21 (5.6%)
**Job Tenure**	0 – 1 year	74 (19.8%)
	2 – 5 years	114 (30.6%)
	6 -10 years	91 (24.4%)
	Over 10 years	94 (25.2%)
**Job Position**	TF-CBT therapists	96 (25.7%)
	Other therapists	231 (61.9%)
	Leaders	46 (12.3%)
**Work Location**	TF-CBT CAMHS ^a^	291 (78%)
	Other CAMHS	82 (22%)

^a^ Child and Adolescent Mental Health Services in which Trauma-Focused Cognitive Behavioral Therapy was implemented

### Measures

A survey package was developed to collect the demographics and self-report data. Number of items used in the measurement of each variable and the reliability measurements (Cronbach's α) are presented in Table 2.

**Table 2:** Prevalence results

		**Levels based on t-scores **^a^		
		**Low**	**Medium**	**High**
**Wellbeing measures** *N* = 344	CS ^b^	26%	49%	25%
	BO ^c^	29%	46%	25%
	STS ^d^	28%	50%	22%
**Attitudes (EBPAS **^**e**^**)** *N* = 373	Total score	27% (< 2.75)	49% (2.75 – 3.30)	24% (= > 3.31)
**Turnover intention** *N* = 256	68% (Scores < 18)		32% medium to high (Scores > 18)	

Levels are generated using quartiles of total scores

^a^ t-scores below 43 and over 57 correspond low and high levels, respectively

^b^ Compassion satisfaction

^c^ Burnout

^d^ Secondary traumatic stress

^e^ *EBPAS* Evidence-based practice attitude scale

#### Demographic questionnaire

Background information that was collected included age, gender, professional background, job tenure (years of working in current CAMHS), and job position (leaders, TF-CBT therapists, and non-TF-CBT therapists). This information was used to test the hypotheses and to compare the two groups of therapists across demographics.

#### The Professional Quality of Life Scale, version 5 (ProQOL-V)

The ProQOL-V [1] is a self-report, 5-point Likert scale with 30 items and the most used measure of the negative (compassion fatigue) and the positive (compassion satisfaction) outcomes of working in helping professions. Compassion fatigue is made up of two subscales that measure burnout and secondary traumatic stress, each with 10 items. Respondents are asked to read each statement, for example, “I believe I can make a difference through my work” and determine how frequently they have experienced it in the last 30 days by choosing one of the 5 alternatives from never (1) to very often (5). t-scores were computed based on instructions given by Stamm [1]. Using t-scores has the advantage that scores from different subscales, ProQOL versions and studies can be comparable. The scales have been found to have a good to excellent reliability (Cronbach alpha ranging from 0.75 to 0.88) and good construct validity [1]. The Norwegian version of The ProQOL-V [59] was used in the present study.

#### Turnover Intention Scale (TIS)

The staff’s intention to leave was measured by the six-item version (TIS-6), which is developed and validated by Bothma and Roodt [10] and has good reliability (α = 0.80) as well as criterion-predictive and differential validity, i.e., it can predict the actual turnover behavior significantly. Participants are asked to read each question, for example, ‘How often have you considered leaving your job?’ and indicate their response by using a 5-point Likert rating scale from ‘never’ (1) to ‘always’ (5). A sum score (from 6 to 30) was computed for each individual where scores 18 to 30 can indicate a middle to a high level of intention to leave. The scale was translated into Norwegian by the research team at the Norwegian Center for Violence and Traumatic Stress Studies using the translate-back-translate method. The turnover intention scale was only administered in CAMHS that participated in the implementation program.

#### Evidence-Based Practice Attitude Scale (EBPAS)

The EBPAS [60, 61] is a well-established 15-item self-report scale that measures attitudes towards the adoption of EBPs on four dimensions: *Appeal* (referring to how much the EBP is intuitively appealing), *Divergence* (perceived divergence between the EBP and current practices), *Requirements* (likelihood of adopting EBP if it is requested by the service, supervisor or by agency mandates), and *Openness* (willingness to try new interventions). Participants indicate the extent to which they agree with each statement on a 5-point Likert scale ranging from 0 (Not at all) to 4 (To a very great extent). After reversing ratings on the divergence subscale, a total mean score was computed on which higher scores indicate more positive attitudes. The Norwegian version of the scale was used in the present study which has good psychometric properties [62].

### Statistical analyses

IBM SPSS Statistics (Version 27.0, IBM Corporation) was used to test the required assumptions and to conduct descriptive and inferential analyses including group comparison *t*-tests, Analysis of Covariance (ANCOVA), One-Way ANOVA, and bivariate correlation analyses to test H1 to H4, respectively. Necessary adjustments were made when the assumptions were not met. Non-parametric tests, i.e., Mann–Whitney U, and Chi-squared, **(**χ^2^) tests were used to examine whether there are significant differences in demographics between the two groups of therapists.

## Results

### Hypothesis 1. Most therapists will report medium to high levels of both compassion fatigue (i.e., burnout and secondary traumatic stress) and compassion satisfaction

Based on t-score cutoffs reported in Stamm ([1] p. 28), the majority of respondents (over 70%) reported medium to high levels (t-scores above 43 and 57, respectively) of both compassion satisfaction, and symptoms of burnout and secondary traumatic stress (Table 2). These results support Hypothesis 1.

We also examined the prevalence rates in each group of therapists (i.e., TF-CBT and others) and found the same pattern of results (i.e., over 70% reported medium to high levels of compassion satisfaction, burnout, and secondary traumatic stress). The prevalence data also showed that one-third (32%) of respondents expressed a medium to high level of turnover intention (sum scores over 18), and that the level of turnover intention was greater among therapists who did not utilize TF-CBT (40%) compared to that among TF-CBT therapists (25%; χ^2^** = **5.12, *p* = 0.02).

### Hypothesis 2. Burnout, secondary traumatic stress, and turnover intention will be related positively to each other and negatively to compassion satisfaction, age, and job tenure

The descriptive and correlation statistics for measured variables are presented in Table 3. Consistent with our hypothesis**,** burnout, secondary traumatic stress, and turnover intention correlated positively with each other and negatively with compassion satisfaction. That is, higher ratings of burnout and secondary traumatic stress were associated with lower compassion satisfaction and higher intention to leave. However, only compassion satisfaction showed a positive, but weak, relationship with age (*r* = 0.14), and job tenure (*r* = 0.10) indicating that older therapists and those with longer job experience in the current CAMHS expressed slightly higher compassion satisfaction.

**Table 3:** Descriptive and correlation statistics for measured variables

Variables	Items (α) ^a^	Mean/ Median (SD) ^b^		Possible Range (Actual Range)	Correlation			
		**TF-CBT** **Group**	**Comparison** **Group**		**CS **^**c**^	**BO **^**d**^	**STS **^**e**^	**TI **^**f**^
**PROQOL: **^**g**^								
**a) CS**	10 (.85)	40.71 (.50)	39.23 (.33)	10 – 50 (25 – 50)	1	-.60**	-.15**	-.29**
**b) BO**	10 (.74)	20.43 (.43)	21.66 (.31)	10 – 50 (10 – 34)	-.60**	1	.46**	.44**
**c) STS**	10 (.76)	19.20 (.47)	19.65 (.30)	10 – 50 (10 – 41)	-.15**	.46**	1	.16*
**TI**	6 (.77)	14.62 (.43)	16.06 (.44)	6 – 30 (6—26)	-.29**	.44**	.16*	1
**EBPAS: **^**h**^								
**a) Appeal**	4 (.71)	3.32 (.05)	3.19 (.04)	0 – 4 (1 – 4)	.15**	-.06	-.03	.15*
**b) Divergence**	4 (.56)	.90 (.05)	.99 (.04)	0 – 4 (1.75 – 4)	.06	.03	.16**	.10
**c) Requirement**	3 (.76)	2.79 (.05)	2.70 (.05)	0 – 4 (.33 – 4)	.17**	-.14**	-.06	.02
**d) Openness**	4 (.77)	3.04 (.05)	2.92 (.04)	0 – 4 (1 – 4)	.21**	-.11*	-.10	.01
**EBPAS total**	15 (.81)	3.07 (.04)	2.95 (.03)	0 – 4 (1.56 – 3.94)	.18**	-.13*	-.12*	.03
**Age**	5 ( -)	40—49	40—49	20 – 60 > (20 – 60 >)	.14**	-.04	-.02	-.03
**Job Tenure**	4 ( -)	2 – 5 years	2 – 5 years	0 – 10 > (0—10 >)	.10*	-.02	-.04	-.07

The median value and the Spearman rank correlation coefficients are presented for age and job tenure because they were ordered in categories

^a^ Cronbach’s alpha

^b^ Standard deviation

^c^ Compassion satisfaction

^d^ Burnout

^e^ Secondary traumatic stress

^f^ Turnover Intention

^g^ Professional Quality of Life Scale

^h^ Evidence-based practice attitude scale

^*^ *p* < .05

^**^ *p* < .01, two-tailed

### Hypothesis 3. On average, TF-CBT trained therapists will report lower ratings of burnout, secondary traumatic stress, and turnover intention along with higher compassion satisfaction than TF-CBT untrained therapists, even after controlling for the effect of therapists’ attitude toward EBPs

We first examined whether the two groups of therapists (TF-CBT therapists vs. non-TF-CBT therapists) were comparable across demographic characteristics. Over 80% of respondents in both groups were females but results from Crosstab’s analysis showed that groups differed a little in proportion of gender categories (χ^2^** = **7.12, *p* = 0.01). That is, a greater proportion of TF-CBT therapists (93%) were females compared to that in the comparison group (females = 81%). Based on results from post hoc Z- test independent proportions, groups were comparable across 8 categories of educational background, and frequencies did not differ significantly except for frequencies in Medicine and Sociology categories in both of which the frequencies were greater in the comparison group (17 & 21, respectively) than in the TF-CBT group (1 & 16), total χ^2^ = 17.07, *p* = 0.048. Mann–Whitney U tests showed that the two therapist groups were also comparable in mean rank of age categories (z = -0.55, *p* = 0.58) and job tenure (z = -1.5, *p* = 1.33). Finally, results revealed a high rate of medium to high level of positive attitudes towards EBPs both among TF-CBT (76%) and *non*-TF-CBT therapists (71%), z** = **-0.85, *p* = 0.39).

Then, independent samples t-tests were used to compare mean scores between TF-CBT and other therapists working in CAMHS. As reported in Table 4, consistent with Hypothesis 3, results showed that TF-CBT therapists reported lower burnout (mean difference, MD =—2.68) and lower turnover intention (MD =—1.44), along with higher compassion satisfaction (MD = 2.91) compared to other therapists (comparison group), even after controlling for the effect of therapists’ attitudes towards EBPs in One-Way ANCOVAs (all *p* values < 0.05). However, the difference between secondary traumatic stress t-scores was not significant (MD = -1.02).

**Table 4:** Summary of inferential testing

	**t / F**	***df***	***p***	***d*** ^a^ ***/*** **η**^**2**^ ^b^	**95% CI** ^c^ **[LL, UL]**
BO _TF-CBT vs. other therapists_	- 2.22	325	.027	-.27	-.51, -.03
STS _TF-CBT vs. other therapists_	-.83	325	.41	-.10	-.34, .14
CS _TF-CBT vs. other therapists_	2.44	325	.015	.30	.06, .53
TI _TF-CBT vs. other therapists_	- 2.34	208	.020	- .32	-.59, -.04

^a^ Cohen’s *d* point estimate effect size < .5 and

^b^ Eta Squared < .06 are defined as small effects [58],

^c^ *CI* Confidence interval of effect sizes

The Levene’s tests showed equality of error variances across therapist groups except in turnover intention measurements (*F* = 5.10; *p* = .025) for which an adjusted *t* value is reported

## Discussion

### Prevalence findings

Results showed that over 70% of the respondents suffer from a medium (~ 48%) to high (~ 24%) level of burnout and secondary traumatic stress symptoms although they also express medium to high level of compassion satisfaction. This feeling of fulfillment may be one of the reasons that the majority of respondents (68%) reported a low level of intention to leave their job in the current or near future. Importantly, we found similar rates both among TF-CBT trained and untrained therapists. This is the first prevalence study from Norwegian CAMHS and indicates that preventive measurements are required for all therapists.

The prevalence of compassion satisfaction among child therapists has not been studied. However, the observed rate in the present study is comparable with compassion satisfaction rate among therapists working with adults [18]. The negative outcomes of working with trauma have been studied in different samples and countries [7, 19, 63–67] and are generally consistent with the present findings. However, the rate of therapists who reported high level of secondary traumatic stress symptoms in our sample (22%) differ from some previous studies in which the same scale was used to measure professional quality of life among therapists working with traumatized *adults, and* found rates either as high as 70% [18] or as low as only 6% [25]. The reasons for these discrepancies are not clear. Some studies have reported between discipline groups differences in wellbeing measures showing for example that psychiatrists and psychologists were comparatively emotionally exhausted [26, 68]. Compared to our study (45%), participants in Sodeke-Gregson et al. [18] included a larger proportion of psychologists (70%), which might have contributed in the higher risk of secondary traumatic stress symptoms reported in their study. A post hoc multivariate analysis showed that psychologists in our study reported higher ratings on burnout and secondary traumatic stress along with lower compassion satisfaction than other professional groups, although this difference was not significant when controlling for multiple testing effects with Bonferroni correction. Future studies should investigate which factors induce potential discipline-related differences in wellbeing measure.

Almost one-third of respondents (32%) in the present study reported a medium to high level of turnover intention, which is consistent with several findings on *actual turnover* rate among mental health and child welfare staff [13, 45, 69–73]. Since turnover intention is a strong cognitive precursor of actual turnover behavior [8, 74], the present findings can be alarming and should be addressed.

### Correlational findings

As expected, results showed moderate to strong relationships (*r* > 0.3) [58] between wellbeing measures and turnover intention, which is consistent with several other studies [27, 30, 31, 36, 75–77]. However, the relationship of compassion satisfaction with burnout (*r* = -0.60) was four times stronger than that with secondary traumatic stress (*r* = -0.15). This may indicate that compassion satisfaction can, indeed, act as a protective factor against negative outcomes of work with trauma and mental illness, especially burnout. Together with prevalence results, our findings are also in line with the notion that the positive and negative measures of wellbeing are related but independent concepts and one can experience high level of both [29]. Moreover, results showed that only compassion satisfaction had a weak positive relationship with age and job tenure. Longer work experiences, maturity, and access to adequate trauma specific training and supervision, may have enhanced clinicians’ therapy and coping skills, and lead to slightly higher perceived self-efficacy and compassion satisfaction.

The strength of the relationship between burnout and secondary traumatic stress in the present study (*r* = 0.46) is large but smaller than that in other studies (weighted *r* = 0.74) with the same type of sample (secondary exposure to trauma), the same scale (i.e., ProQOL), and the same theoretical approach (compassion fatigue framework) to operationalize burnout and secondary traumatic stress [37]. This discrepancy can be related to other factors that moderate the strength of relationship between these concepts like sociocultural and linguistic differences. Sociocultural context can influence the outcomes of work-related trauma exposure by influencing emotional processing and shaping emotional experiences and through differences in organizational resources, polices and characteristics [37, 38].

Finally, correlational analysis showed that turnover intention had a stronger relationship with burnout (*r* = 0.44) than with compassion satisfaction (*r* = -0.29) and secondary traumatic stress (*r* = 0.16). That is, although most clinicians expressed medium to high level of secondary trauma stress symptoms, experiences of secondary traumatic stress had a weak association with turnover intention. These results are consistent with the job demands-resources model [48] and previous findings showing that burnout and job engagement/ satisfaction mediate the relationship between job demands, job resources, and turnover intention [78–81]. Moreover, considering the ProQOL-V items by which these constructs are measured, the results indicate that feelings of being “unproductive, or overwhelmed, bogged down, worn out, and trapped” by the job or the system is associated more strongly with intent to leave than feelings of being “unsatisfied, unhappy, incompetent, or unmotivated”. Therefore, our results suggest that effective measurements to reduce burnout are especially important for staff retention in CAMHS.

### Between group differences

On average, TF-CBT trained therapists, compared to untrained therapists, reported a significant lower degree of burnout and turnover intention along with higher compassion satisfaction, both before and after controlling for variations in therapists’ attitudes towards EBPs. TF-CBT therapists also reported numerically lower symptoms of secondary traumatic stress than the comparison group, but mean differences did not reach the significance level. The findings are consistent with both self-efficacy and job demands-resources theories and with findings from some other studies that have shown an association between the use of EBPs and higher compassion satisfaction [25] or reduced risk of burnout [25, 35, 41], and turnover intention and behavior [9, 40]. Yet, other studies did not find a positive effect of using EBPs on professional wellbeing [23], burnout [11] or team turnover rates in mental health services [13, 45], and some therapists have expressed concerns that using EBPs may reduce the level of engagement between client and therapists [82] or increase the workload, stress and staff turnover [39, 42].

These discrepancies raise the question that which factors are associated with a positive effect of training in EBPs on therapist’s wellbeing and retention intention. Some researchers have found that therapists’ attitudes toward EBPs can play a role [11, 44, 45]. We also found that positive attitudes towards EBPs were associated with higher compassion satisfaction, and lower level of burnout and secondary traumatic stress. However, since most therapists in both groups expressed a medium to high level of positive attitude toward EBPs, other factors proposed by the self-efficacy theory and the job demands-resources model might have contributed in the improved professional wellbeing and turnover intention among TF-CBT trained therapists. According to the job demands-resources theory [48], job and personal resources like access to trauma-specific trainings and the sense of mastery and self-efficacy can either buffer the negative effects of job demands and reduce job strain [33] or facilitate achieving occupational goals by increasing work engagement [49]. Empirical studies, also from Norwegian CAMHS [83] have shown that TF-CBT is indeed highly effective, and a positive association of higher self-efficacy with both training in TF-CBT [54] and higher levels of compassion satisfaction [82] have been recently reported. Although perceived self-efficacy is not measured in the present study, the ProQOL-V items by which compassion satisfaction is measured in the present study include statements that can reflect therapists’ perceived level of self-efficacy (e.g., “I am pleased with how I am able to keep up with professional updates”).

When implemented successfully, training in TF-CBT can also provide other job resources like strategic planning, necessary boundaries, and control through predefined and structured treatment sessions, along with proper supervisory and administrative support, and building strong teams that are devoted, supportive and open to give and accept constructive feedback which are associated with more positive attitudes towards EBPs [38, 43]. Higher self-beliefs can reciprocally lead to positive attitudes and openness towards learning and mastering new practices. Adequate training and supervision in structured, multifaceted EBPs like TF-CBT can then enhance clinicians’ skills and perceived competency and positive attitudes [43, 54]. This increase in perceived self-efficacy will be reinforced when using TF-CBT becomes accompanied with actual improvements in traumatized children. Since helping children can be emotionally and juridically complex and demanding [21, 22], even small progress after using TF-CBT can possibly be highly satisfactory and increase therapist’s perceived self-efficacy, wellbeing, and retention intentions. However, The positive effects of using TF-CBT and potentially other EBPs can be expected when it fits the needs of target clients and mental health providers and is adopted in a way to be culturally compatible [84]. Further studies are required to disentangle the factors associated with the protective effect of using TF-CBT among mental health providers, and to investigate TF-CBT versus other evidence-based treatments.

### Theoretical and practical implications

Theoretically, the present results suggest that training in TF-CBT can increase the available job and personal resources and be associated with better professional quality of life and an increased tendency to stay at the current job among therapists working with children. Future studies should investigate whether training in TF-CBT is associated with higher ratings on e.g., perceived social support, autonomy, or role clarity as job resources.

The present study has several practical implications. First, the observed higher level of wellbeing and retention intention among TF- CBT therapists imply that training in TF-CBT is cost effective, which is an organizationally important information both for the current clinics that have implemented TF-CBT, and for other therapists and decision makers who are considering joining or initiating implementation programs with well-established EBPs in general, and TF-CBT in specific. Moreover, the prevalence findings are alarming and call for taking adequate immediate and long-term measurements, both to improve the cost-effectiveness and the sustainability of the implementation program, and to prevent turnover among trained staff [13, 14]. Such interventions should be based on empirical findings relevant to professional wellbeing in CAMHS. For example, a recent study [85] found potential benefits from combining a self-care approach, referred to as “PRACTICE What You Preach” into TF-CBT training for reducing level of secondary traumatic stress. Although therapist directed interventions like this or mindfulness training can help, previous studies have shown that organizational interventions are more alterable and are more efficient in reducing burnout and increase personal efficacy [6]. Work-related measurements can include promoting and facilitating trauma training and utilization of EBPs; reducing case overloads, role conflict and ambiguity; increasing possibilities for job autonomy, social support, and supervision, adapting regular screening systems for early detection of individuals at risk, as well as reforming the organizational climate and the leadership style [25, 36, 38, 73, 86]. However, as mentioned, focusing on factors associated with burnout should receive high priority. For example, based on burnout items (e.g., I feel connected to others), improving the quality of supervision and teamwork aspects of training in TF-CBT could be effective in reducing burnout level. However, future studies should also investigate which specific job resources (work-, organization-, and implementation-related factors) and elements of TF-CBT that are associated with higher level of compassion satisfaction and lower risk of burnout and turnover intention observed among TF-CBT therapists.

### Study strength and limitations, and suggestions for future studies

The present study replicates some previous findings and expands the literature by filling the research gap in two areas, i.e., to the best of our knowledge, it presents the first prevalence data on compassion satisfaction and turnover intention among mental health professionals working in CAMHS, and it provides the first evidence for the potential positive effect of training in TF-CBT on both professional quality of life (i.e., compassion satisfaction and fatigue) and turnover intention. The study has sufficient power, the variables are measured by well-established scales, and the observed effect sizes are in line with previous findings in this field and, generally, in the field of psychology [87].

However, there are also limitations that should be kept in mind when interpreting the results. First, the cross-sectional design of this study inhibits causality interpretations. Therefore, before making any firm conclusions, these findings should be replicated in larger samples, preferably in longitudinal studies with objective behavioral (i.e., register data on sick leave) or psychophysiological measures of stress or satisfaction (i.e., plasma catecholamines and heart rate variability). A relatively low respondent rate is also worth mentioning, although a respondent rate of 30% is comparable with the rate of participation among mental health therapists [88, 89], and according to the registered data [90], our sample demographics are representative of professionals working in Norwegian CAMHS.

Finally, it is also noteworthy that there was some discrepancy in the prevalence data when the raw score cutoffs of ProQOL-V scale ([1] p. 28) were used (i.e., the rates of high risks for burnout and secondary traumatic stress dropped to zero). A recent study [91] on psychometric evaluation of the ProQOL-V reported that there may be an error in raw score cutoffs for individual scoring. Considering this point and to make comparisons between samples and studies possible, we used t-scores in the analyses. In any case, it should be considered that prevalence findings are probably an underestimation of true rates because individuals with highly poor wellbeing are expectedly either on sick leave, or have already left the job, or may not be motivated to participate. In general, these prevalence findings should be interpreted cautiously because the ProQOL-V is not made for diagnostic purposes [1]. Nevertheless, it is a suitable tool for wellbeing screening and planning supportive and preventive actions to improve the professional quality of life. Future studies should investigate which common and specific factors at clients, therapist and organizational levels are involved in developing or preventing of negative and positive outcomes among trauma therapists. The impact of cultural and individual difference factors on how using EBPs affect therapists should not be ignored.

## Conclusion

Working with traumatized and distressed children can have both negative and positive outcomes for the wellbeing and professional quality of life among therapists which can in turn influence staff retention and the quality of provided care. The present study provides the first empirical evidence for a potential positive effect of training in TF-CBT on therapists. That is, TF-CBT trained therapists scored lower on burnout and turnover intention and higher on compassion satisfaction compared to TF-CBT untrained therapists working in Norwegian CAMHS. These findings are relevant for public health planning. The results furthermore demonstrated medium to high levels of burnout and secondary traumatic stress in both groups of therapists, hence proper interventions should be taken into consideration by decision makers. The success and sustainability of EBPs is not guaranteed unless the subjective wellbeing of trained clinicians is taken seriously, and they are willing to stay in their job.

### Availability of data and material

The datasets generated and/or analyzed during the current study are not publicly available due to their proprietary nature but are available from the corresponding author on reasonable request.

## Data Availability

The datasets will be available from the corresponding author on reasonable request.

## Abbreviations

CAMHS: Child and Adolescent Mental Health Services
TF-CBT: Trauma-Focused Cognitive Behavioral Therapy
PTSD: Post-traumatic stress disorder
EBP: Evidence-based practices
EBPAS: Evidence-Based Practice Attitude Scale
ProQOL-V: The Professional Quality of Life Scale, version 5

## References

[1] Stamm BH. The Concise ProQOL Manual. 2nd Edition, Pocatello, ID: ProQOL.org; 2010.

[2] Danna K, Griffin RW (1999). Health and well-being in the workplace: a review and synthesis of the literature. J Manag.

[3] Linley PA, Joseph S (2007). Therapy work and therapists' positive and negative well-being. J Soc Clin Psychol.

[4] Turley R, Roberts S, Foster C, Warner N, El-Banna A, Evans R (2022). Staff wellbeing and retention in children’s social work: systematic review of interventions. Res Soc Work Pract.

[5] Cheung CK, Chow EOW (2011). Reciprocal influences between burnout and effectiveness in professional care for elders. Soc Work Health Care.

[6] Johnson J, Hall LH, Berzins K, Baker J, Melling K, Thompson C (2018). Mental healthcare staff well-being and burnout: a narrative review of trends, causes, implications, and recommendations for future interventions. Int J Ment Health Nurs.

[7] Morse G, Salyers MP, Rollins AL, Monroe-DeVita M, Pfahler C (2012). Burnout in mental health services: a review of the problem and its remediation. Adm Policy Ment Health.

[8] Tett RP, Meyer JP (1993). Job satisfaction, organizational commitment, turnover intention, and turnover: path analyses based on meta-analytic findings. Pers Psychol.

[9] Aarons GA, Sommerfeld DH, Hecht DB, Silovsky JF, Chaffin MJ (2009). The impact of evidence-based practice implementation and fidelity monitoring on staff turnover: evidence for a protective effect. J Consult Clin Psychol.

[10] Bothma CFC, Roodt G. The validation of the turnover intention scale. SA J Human Resour Manag. 2013;11:1–12.

[11] Kim JJ, Brookman-Frazee L, Gellatly R, Stadnick N, Barnett ML, Lau AS (2018). Predictors of burnout among community therapists in the sustainment phase of a system-driven implementation of multiple evidence-based practices in children's mental health. Prof Psychol Res Pract.

[12] Garner BR, Hunter BD, Modisette KC, Ihnes PC, Godley SH (2012). Treatment staff turnover in organizations implementing evidence-based practices: turnover rates and their association with client outcomes. J Subst Abuse Treat.

[13] Woltmann EM, Whitley R, McHugo GJ, Brunette M, Torrey WC, Coots L (2008). The role of staff turnover in the implementation of evidence-based practices in mental health care. Psychiatr Serv.

[14] Swain K, Whitley R, McHugo GJ, Drake RE (2010). The sustainability of evidence-based practices in routine mental health agencies. Community Ment Health J.

[15] Aarons GA (2005). Measuring provider attitudes toward evidence-based practice: consideration of organizational context and individual differences. Child Adolesc Psychiatr Clin N Am.

[16] Cooper JL, Aratani Y (2009). The status of states’ policies to support evidence-based practices in children’s mental health. Psychiatr Serv.

[17] ISTSS Posttraumatic Stress Disorder Prevention and Treatment Guidelines: Methodology and Recommendations. IL, USA: International Society for Traumatic Stress Studies. 2019. Available online: www.istss.org.

[18] Sodeke-Gregson EA, Holttum S, Billings J. Compassion satisfaction, burnout, and secondary traumatic stress in UK therapists who work with adult traumaclients. Eur J Psychotraumatol. 2013;4. 10.3402/ejpt.v4i0.21869. 10.3402/ejpt.v4i0.21869 PMC3877781 24386550

[19] Newell JM, MacNeil GA (2011). A comparative analysis of burnout and professional quality of life in clinical mental health providers and health care administrators. J Work Behav Health.

[20] Bride BE (2007). Prevalence of secondary traumatic stress among social workers. Soc Work.

[21] Korkeila JA, Töyry S, Kumpulainen K, Toivola JM, Räsänen K, Kalimo R (2003). Burnout and self-perceived health among Finnish psychiatrists and child psychiatrists: a national survey. Scand J Public Health.

[22] de Figueiredo S, Yetwin A, Sherer S, Radzik M, Iverson E (2014). A cross-disciplinary comparison of perceptions of compassion fatigue and satisfaction among service providers of highly traumatized children and adolescents. Traumatology.

[23] van Minnen A, Keijsers GP (2000). A controlled study into the (cognitive) effects of exposure treatment on trauma therapists. J Behav Ther Exp Psychiatry.

[24] McNicholas F, Sharma S, Oconnor C, Barrett E (2020). Burnout in consultants in child and adolescent mental health services (CAMHS) in Ireland: a cross-sectional study. BMJ Open.

[25] Craig CD, Sprang G (2010). Compassion satisfaction, compassion fatigue, and burnout in a national sample of trauma treatment therapists. Anxiety Stress Coping.

[26] Rossi A, Cetrano G, Pertile R, Rabbi L, Donisi V, Grigoletti L (2012). Burnout, compassion fatigue, and compassion satisfaction among staff in community-based mental health services. Psychiatry Res.

[27] Salloum A, Kondrat DC, Johnco C, Olson KR (2015). The role of self-care on compassion satisfaction, burnout and secondary trauma among child welfare workers. Child Youth Serv Rev.

[28] Stamm BH. Measuring compassion satisfaction as well as fatigue: Developmental history of the Compassion Satisfaction and Fatigue Test. In C. R. Figley (Ed.), Treating compassion fatigue. Brunner-Routledge; 2002. p. 107–119. https://psycnet.apa.org/record/2002-17425-005.

[29] Huppert FA, Whittington JE (2003). Evidence for the independence of positive and negative well-being: implications for quality of life assessment. Br J Health Psychol.

[30] Scanlan JN, Still M (2019). Relationships between burnout, turnover intention, job satisfaction, job demands and job resources for mental health personnel in an Australian mental health service. BMC Health Serv Res.

[31] Yanchus NJ, Periard D, Osatuke K (2017). Further examination of predictors of turnover intention among mental health professionals. J Psychiatr Ment Health Nurs.

[32] Collins S, Long A (2003). Working with the psychological effects of trauma: consequences for mental health-care workers–a literature review. J Psychiatr Ment Health Nurs.

[33] Tremblay MA, Messervey. The Job Demands-Resources Model: Further Evidence for the Buffering Effect of Personal Resources. SA J Ind Psychol. 2011;37:10-19.

[34] Cetrano G, Tedeschi F, Rabbi L, Gosetti G, Lora A, Lamonaca D (2017). How are compassion fatigue, burnout, and compassion satisfaction affected by quality of working life? Findings from a survey of mental health staff in Italy. BMC Health Serv Res.

[35] Aarons GA, Fettes DL, Flores LE, Sommerfeld DH (2009). Evidence-based practice implementation and staff emotional exhaustion in children’s services. Behav Res Ther.

[36] Singh J, Karanika-Murray M, Baguley T, Hudson J (2020). A systematic review of job demands and resources associated with compassion fatigue in mental health professionals. Int J Environ Res Public Health.

[37] Cieslak R, Shoji K, Douglas A, Melville E, Luszczynska A, Benight CC (2014). A meta-analysis of the relationship between job burnout and secondary traumatic stress among workers with indirect exposure to trauma. Psychol Serv.

[38] Voss Horrell SC, Holohan DR, Didion LM, Vance GT (2014). Treating traumatized OEF/OIF veterans: how does trauma treatment affect the clinician?. Prof Psychol Res Pract.

[39] Nelson TD, Steele RG, Mize JA (2006). Practitioner attitudes toward evidence-based practice: themes and challenges. Adm Policy Ment Health.

[40] Rollins AL, Salyers MP, Tsai J, Lydick JM (2010). Staff turnover in statewide implementation of ACT: relationship with ACT fidelity and other team characteristics. Adm Policy Ment Health.

[41] Wilkinson CB, Infantolino ZP, Wacha-Montes A (2017). Evidence-based practice as a potential solution to burnout in university counseling center clinicians. Psychol Serv.

[42] Skriner LC, Wolk CB, Stewart RE, Adams DR, Rubin RM, Evans AC, Beidas RS (2018). Therapist and organizational factors associated with participation in evidence-based practice initiatives in a large urban publicly funded mental health system. J Behav Health Serv Res.

[43] Barnett M, Brookman-Frazee L, Regan J, Saifan D, Stadnick N, Lau A (2017). How intervention and implementation characteristics relate to community therapists’ attitudes toward evidence-based practices: a mixed methods study. Adm Policy Ment Health.

[44] Beidas RS, Marcus S, Aarons GA, Hoagwood K, Schoenwald S, Evans A, Mandell DS (2015). Individual and organizational factors related to community clinicians’ use of therapy techniques in a large public mental health system. JAMA Pediatr.

[45] Beidas RS, Marcus S, Wolk CB, Powell B, Aarons GA, Evans AC (2016). A prospective examination of clinician and supervisor turnover within the context of implementation of evidence-based practices in a publicly funded mental health system. Adm Policy Ment Health.

[46] Bandura A (2010). Self-Efficacy. The Corsini Encyclopedia of Psychology, American Cancer Society.

[47] Bandura A (1977). Self-efficacy: toward a unifying theory of behavioral change. Psychol Rev.

[48] Schaufeli WB, Taris TW (2014). A critical review of the job demands-resources model: Implications for improving work and health. Bridging occupational, organizational and public health.

[49] Xanthopoulou D, Bakker AB, Demerouti E, Schaufeli WB (2007). The role of personal resources in the job demands–resources model. Int J Stress Manag.

[50] Cohen J, Mannarino AP (2008). Disseminating and implementing trauma-focused CBT in community settings. Trauma Violence Abuse.

[51] Watkins LE, Sprang KR, Rothbaum BO (2018). Treating PTSD: a review of evidence-based psychotherapy interventions. Front Behav Neurosci.

[52] Allen B, Johnson JC (2012). Utilization and implementation of trauma-focused cognitive-behavioral therapy for the treatment of maltreated children. Child Maltreat.

[53] Thomas FC, Puente-Duran S, Mutschler C, Monson CM (2020). Trauma-focused cognitive behavioral therapy for children and youth in low and middle-income countries: a systematic review. Child Adolesc Ment Health.

[54] Becker-Haimes EM, Wislocki K, DiDonato S, Jensen-Doss A (2021). Predictors ofclinician-reported self-efficacy in treating trauma-exposed youth. J Trauma Stress.

[55] Skar AS, Ormhaug SM, Jensen TK (2019). Reported levels of upset in youth after routine trauma screening at mental health clinics. JAMA Netw Open.

[56] Fagermoen EM, Østensjø T, Skagemo CU, Moen GM, Husebø GK (2017). Traumefokusert kognitiv atferdsterapi og komplekse traumer. Tidsskrift for Norsk Psykologforening.

[57] Faul F, Erdfelder E, Buchner A, Lang A-G (2009). Statistical power analyses using G*Power 3.1: tests for correlation and regression analyses. Behav Res Methods.

[58] The CJ, Size E (1988). Statistical power analysis for the behavioral sciences.

[59] Lauvrud C, Nonstad K, Palmstierna TO (2009). Occurence of post-traumatic stress symptoms and their relationship to profession-al quality of lilfe (ProQOL) in nursing staff at a forensic psychiatric security unit: a cross sectional study. Health Qual LifeOutc.

[60] Aarons GA (2004). Mental health provider attitudes toward adoption of evidence-based practice: the Evidence-Based Practice Attitude Scale (EBPAS). Ment Health Serv Res.

[61] Aarons GA, Glisson C, Hoagwood K, Kelleher K, Landsverk J, Cafri G (2010). Psychometric properties and U.S. National norms of the Evidence-Based Practice Attitude Scale (EBPAS). Psychol Assess.

[62] Egeland KM, Ruud T, Ogden T, Lindstrøm JC, Heiervang KS (2016). Psychometric properties of the Norwegian version of the Evidence-Based Practice Attitude Scale (EBPAS): to measure implementation readiness. Health Res Policy Syst.

[63] Baum N, Moyal S (2020). Impact on therapists working with sex offenders: a systematic review of gender findings. Trauma Violence Abuse.

[64] Cornille TA, Meyers TW. Secondary traumatic stress among child protective service workers: Prevalence, severity and predictive factors. Traumatology. 1999;5:15–31.

[65] Lee HJ, Lee M, Jang SJ (2021). Compassion satisfaction, secondary traumatic stress, and burnout among nurses working in trauma centers: a cross-sectional study. Int J Environ Res Public Health.

[66] Meldrum L, King R, Spooner D. Secondary traumatic stress in case managers working in community mental health services. In: Figley CR, editor. Treating compassion fatigue. Brunner-Routledge; 2002. p. 85–106.

[67] Sage CAM, Brooks SK, Greenberg N (2018). Factors associated with Type II trauma in occupational groups working with traumatised children: a systematic review. J Ment Health.

[68] Onyett S, Pillinger T, Muijen M (1977). Job satisfaction and burnout among members of community mental health teams. J Ment Health.

[69] Aarons G, Sawitzky A (2006). Organizational climate mediates the effect of culture on work attitudes and turnover in mental health services. Adm Policy Ment Health.

[70] Dishop CR, Green AE, Torres E, Aarons GA (2019). Predicting turnover: the moderating effect of functional climates on emotional exhaustion and work attitudes. Community Ment Health J.

[71] Glisson C, Dukes D, Green P (2006). The effects of the ARC organizational intervention on caseworker turnover, climate, and culture in children's service systems. Child Abuse Negl.

[72] Moses TC. Improving Staff Retention in Adolescent Psychiatric Residential Treatment Facilities. Doctoral thesis, Walden University. 2021.

[73] Turley R, Roberts S, Foster C, Warner N, El-Banna A, Evans R (2020). Promoting the retention, mental health and wellbeing of child and family social workers: a systematic review of Workforce interventions.

[74] Knudsen HK, Ducharme LJ, Roman PM (2007). Research participation and turnover intention: an exploratory analysis of substance abuse counselors. J Subst Abuse Treat.

[75] Griffeth RW, Hom PW, Gaertner S (2000). A meta-analysis of antecedents and correlates of employee turnover: update, moderator tests, and research implications for the next millennium. J Manag.

[76] Schaufeli WB, Bakker AB (2004). Job demands, job resources, and their relationship with burnout and engagement: a multi-sample study. J Organ Behav.

[77] Hinderer KA, VonRueden KT, Friedmann E, McQuillan KA, Gilmore R, Kramer B, Murray M (2014). Burnout, compassion fatigue, compassion satisfaction, and secondary traumatic stress in trauma nurses. J Trauma Nurs.

[78] Hoonakker P, Carayon P, Korunka C (2013). Using the Job-Demands-Resources model to predict turnover in the information technology workforce: general effects and gender differences. Psihološka Obzorja / Horizons of Psychology.

[79] Kaiser S, Patras J, Adolfsen F, Richardsen AM, Martinussen M. Using the job demands-resources model to evaluate work-related outcomes among Norwegian health care workers. SAGE Open. 2020;10(3):2158244020947436.

[80] Russell MB, Attoh PA, Chase T, Gong T, Kim J, Liggans GL. Examining burnout and the relationships between job characteristics, engagement, and turnover intention among U.S. educators. SAGE Open. 2020;10(4):2158244020972361.

[81] Wang H, Jin Y, Wang D, Zhao S, Sang X, Yuan B (2020). Job satisfaction, burnout, and turnover intention among primary care providers in rural China: results from structural equation modeling. BMC Fam Pract.

[82] Cicognani E, Pietrantoni L, Palestini L, Prati G (2009). Emergency workers’ quality of life: the protective role of sense of community, efficacy beliefs and coping strategies. Soc Indic Res.

[83] Jensen TK, Holt T, Ormhaug SM, Egeland K, Granly L, Hoaas LC, Wentzel-Larsen T (2013). A randomized effectiveness study comparing trauma-focused cognitive behavioral therapy with therapy as usual for youth. J Clin Child Adolesc Psychol.

[84] Naeem F (2019). Cultural adaptations of CBT: a summary and discussion of the special issue on cultural adaptation of CBT. Cogn Behav Ther.

[85] Deblinger E, Pollio E, Cooper B, Steer RA (2020). Disseminating trauma-focused cognitive behavioral therapy with a systematic self-care approach to addressing secondary traumatic stress: practice what you preach. Community Ment Health J.

[86] Turgoose D, Maddox L (2017). Predictors of compassion fatigue in mental health professionals: a narrative review. Traumatology.

[87] Lovakov A, Agadullina ER (2021). Empirically derived guidelines for effect size interpretation in social psychology. Eur J Soc Psychol.

[88] Cashel ML (2002). Child and adolescent psychological assessment: Current clinical practices and the impact of managed care. Prof Psychol Res Pract.

[89] Hawley KM, Cook JR, Jensen-Doss A (2009). Do noncontingent incentives increase survey response rates among mental health providers? A randomized trial comparison. Adm Policy Ment Health.

[90] Statistics Norway. https://www.ssb.no/statbank/table/09550/. Accessed 07 Apr 2022.

[91] Hagan JL. Psychometric evaluation of the ProQOL version 5 for assessing compassion satisfaction, burnout and secondary traumatic stress in nurses. Int J Nurs Stud. 2019;4(3):60.

